# Skeletal Class III malocclusion treatment using mandibular and maxillary skeletal anchorage and intermaxillary elastics: a case report

**DOI:** 10.1590/2177-6709.24.5.052-059.oar

**Published:** 2019

**Authors:** Mehrnaz Fakharian, Erfan Bardideh, Mostafa Abtahi

**Affiliations:** 1 Torbat Heydarieh University of Medical Sciences, Department of Orthodontics (Torbat Heydarieh, Iran).; 2 Mashhad University of Medical Sciences, School of Dentistry, Postgraduate Program in Orthodontics (Mashhad, Iran).; 3 Mashhad University of Medical Sciences, Dental Research Center, Department of Orthodontics (Mashhad, Iran).

**Keywords:** Jaw abnormalities, Retrognathia, Maxillary retroposition, Functional orthodontic appliance, Bone plates.

## Abstract

**Introduction:**

Skeletal Class III malocclusion is one of the most challenging malocclusions to treat. In around 40% of Class III patients, maxillary retrognathia is the main cause of the problem and in most patients, orthopedic/surgical treatments includes some type of maxillary protraction.

**Objective:**

The aim of this case report was to describe a treatment method for a patient with maxillary retrognathia and Class III skeletal discrepancy using mandibular and maxillary skeletal anchorage with intermaxillary elastics.

**Case report:**

A 13-year-old boy with maxillary retrognathia and mandibular prognathism was treated using bilateral miniplates. Two miniplates were inserted in the mandibular canine area and two other miniplates were placed in the infrazygomatic crests of the maxilla. Class III intermaxillary elastics were used between the miniplates.

**Results:**

After eight months of orthopedic therapy, ANB angle increased by 4.1 degrees and ideal overjet and overbite were achieved. Mandibular plane angle was increased by 2.1 degrees and the palatal plane was rotated counterclockwise by 4.8 degrees.

**Conclusion:**

This case showed that the skeletal anchorage treatment method may be a viable option for treating patients with Class III skeletal malocclusion.

## RESUMO

**Introdução:**

a Classe III esquelética é considerada uma das más oclusões mais difíceis de se tratar. Em cerca de 40% dos pacientes afligidos por ela, a principal causa do problema é o retrognatismo maxilar e, na maioria desses pacientes, o tratamento ortopédico/cirúrgico inclui algum tipo de protração da maxila.

**Objetivo:**

o objetivo do presente relato de caso é descrever o método de tratamento de um paciente Classe III com discrepância esquelética e retrognatismo maxilar, usando elásticos intermaxilares e ancoragem esquelética superior e inferior.

**Relato de caso:**

paciente de 13 anos de idade, com retrognatismo maxilar e prognatismo mandibular, tratado com miniplacas inseridas bilateralmente. Duas miniplacas foram instaladas na região dos caninos inferiores, e outras duas miniplacas foram inseridas na região da crista infrazigomática maxilar. Elásticos intermaxilares de Classe III foram conectados às miniplacas.

**Resultados:**

após 8 meses de tratamento ortopédico, o ângulo ANB aumentou 4,1^o^ e obteve-se sobressaliência e sobremordida ideais. O ângulo do plano mandibular aumentou 2,1^o^ e o plano palatal girou 4,8^o^ no sentido anti-horário.

**Conclusão:**

esse caso clínico demonstrou que o método de tratamento com ancoragem esquelética pode ser uma opção válida para pacientes com má oclusão de Classe III esquelética.

## INTRODUCTION

Skeletal Class III malocclusion is one of the most challenging malocclusions to treat. Skeletal Class III discrepancies can be caused by maxillary retrognathia and/or mandibular protrusion.^1^ In around 40% of Class III patients, maxillary retrognathia is the main cause of the problem and in most patients, orthopedic/surgical treatments include some type of maxillary protraction.^2^ ^,^ ^3^ The use of orthopedic force by extraoral traction for protraction of maxillary deficient patients began in the 1970s.^4^ Problems with the growth modification devices in this era were the dental anchorage systems and patients’ compliance.^5^ ^,^ ^6^ In the following years, use of skeletal anchorage (miniplates and miniscrews) and facemask was studied, and unintentional tooth movements were eliminated.^7^ ^-^ ^9^ Still, the biggest problem with the orthopedic treatment of maxillary retrognathia - patient compliance - was not addressed. In 2009, De Clerk proposed using miniplates in maxilla and mandible with intermaxillary elastics for maxillary protraction. With bone-anchored maxillary protraction (BAMP) treatment protocol, patient compliance was a lot higher, and bone-borne traction force could be applied 24 hours per day.^10^ ^-^ ^13^ Thus, the aim of this case report is to describe a treatment method for a patient with maxillary retrognathia and Class III skeletal discrepancy using mandibular and maxillary skeletal anchorage with intermaxillary elastics.

## CASE REPORT

**Figure 1:**
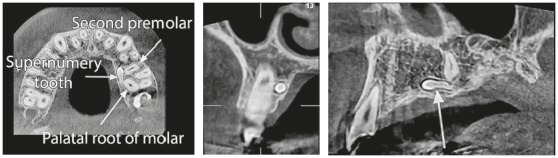
Supernumerary tooth and its position in relation to the adjacent teeth.

A 13-year-old boy with reverse overjet and maxillary retrognathia was referred for orthodontic therapy. Patient’s medical history showed no problems, and no systematic disease that would interfere with orthodontic treatment could be found. The patient had a missing upper left second molar and a supernumerary tooth that was present palatal to the upper left first molar and second premolar (Fig 1). The supernumerary tooth would not affect orthodontic treatment, and a decision was made to not extract the tooth until completion of treatment.

**Figure 2:**
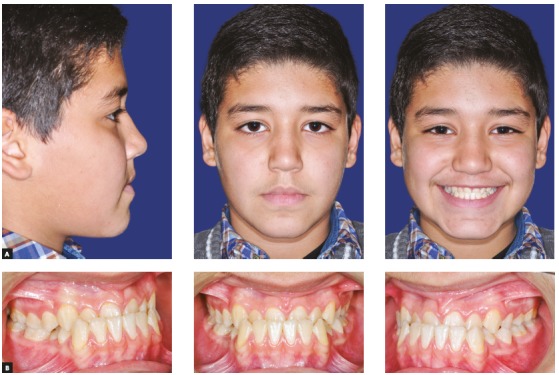
Pretreatment photographs of patient. A) Extraoral: profile; frontal at rest; frontal smiling. B) Intraoral: right side; frontal; left side.

**Figure 3:**
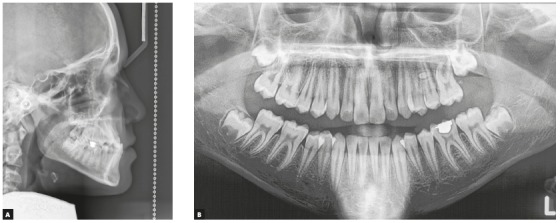
A) Pretreatment lateral cephalometric radiograph. B) Pretreatment panoramic radiograph.

Clinical examination depicted a skeletal Class III malocclusion with a straight profile, retrognathic maxilla and upper lip (Fig 2A). The patient had an anterior crossbite with -2mm overjet and both canines and molars had a Class III dental relationship (Fig 2B). Upon guiding the patient towards the centric relation, the patient had close to an edge to edge anterior occlusion (overjet: -0.5 mm) and the reverse overjet, at least partially was caused by an anterior shift of incisors by the patient. There was a size discrepancy between the anterior maxillary and mandibular teeth (anterior Bolton analysis: 81.7%). The cephalometric analysis also confirmed the Class III malocclusion with maxillary retrognathia as the main cause (SNA=79.9◦, SNB=82.3◦, ANB=-2.5◦). Vertical relations were normal (GoGn-SN=31.9◦, FMA=26.8◦) and the patient had proclined maxillary and retroclined mandibular incisors (U1 to SN=105.3◦ and IMPA=83.9◦) (Fig 3A, Fig 10, Table 1).

**Table 1:** Cephalometric analysis of the patient in pretreatment (T0), postorthopedic (T1), and final post treatment (T2) periods.

Cephalometric parameter	T_0_	T_1_	T_2_	Norm
SNA (degrees)	79.9	82.2	82.2	82.0
SNB (degrees)	82.3	80.7	81.4	80.4
ANB (degrees)	-2.5	1.6	0.8	1.6
Wits (mm)	-11.1	-2.3	-1.2	-1.0
SN - GoGn (degrees)	31.9	34.0	32.1	32.9
FMA (MP-FH) (degrees)	26.8	29.4	28.1	26.2
P-A Face Height (S-Go/N-Me) (%)	64.4	62.5	64.5	65
Palatal plane inclination (degrees)	3.8	-1.0	0.1	-1
Mand plane - Occ plane (degrees)	19.3	18.3	17.1	16.6
Palatal plane - Occ plane (PP-OP) (degrees)	10.8	12.1	11.2	10
U1 . FH (degrees)	110.0	110.4	115.2	111.0
U1 . SN (degrees)	105.3	106.5	109.7	102.6
U1 . NA (degrees)	25.4	26.3	28.4	22.8
U1 - NA (mm)	3.2	4.1	5.7	4.3
U1 - Palatal plane (degrees)	107.8	111.4	114.8	112
L1 - NB (mm)	3.0	4.4	4.2	4
L1 - NB (degrees)	20.1	20.6	25.4	25.3
IMPA (L1-MP) (degrees)	83.9	86.7	92.5	90
FMIA (L1-FH) (degrees)	69.9	61.9	59.3	64.2
Interincisal angle (U1-L1) (degrees)	139.9	131.5	128.6	130
Upper Lip to E-Plane (mm)	-4.2	-2.8	-2.7	-4.7
Lower Lip to E-Plane (mm)	-0.1	-1.9	-1.3	-2

Our treatment objective was to correct the maxillary-mandibular discrepancy by maxillary advancement to eliminate the reverse overjet and to attain an ideal overbite and overjet. Different treatment plans were suggested for achieving the treatment objectives: Use of extra-oral appliance (Facemask), deferring treatment and then performing orthognathic surgery after the completion of growth; or to use miniplates and intermaxillary elastics to protract maxilla.

### Treatment progress

**Figure 4:**
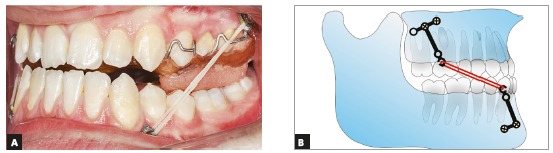
A) Intraoral photograph of plates and intermaxillary elastic. B) Schematic image of plates and elastic.

**Figure 5:**
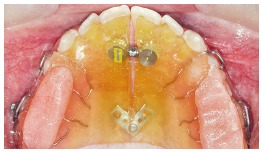
Removable appliance in the maxillary arch.

**Figure 6:**
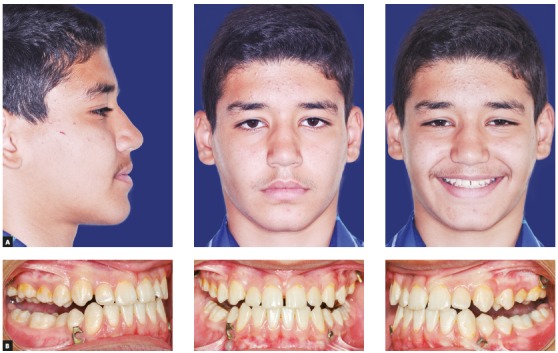
Post-orthopedic treatment photographs. A) Extraoral: profile; frontal at rest; frontal smiling. B) Intraoral: right side; frontal; left side.

**Figure 7:**
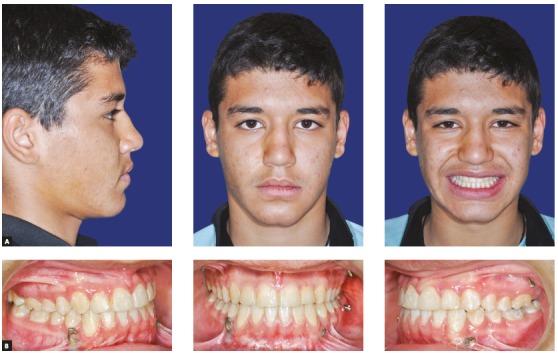
Final post-treatment photographs of patient. A) Extraoral: Profile; frontal at rest; frontal smiling. B) Intraoral: right side; frontal; left side.

Two pairs of bilateral orthodontic miniplates (CMF miniplate; Ortho Select GmbH Implant Technology, Wurmlingen, Germany) were placed in the canine area of the mandible and infrazygomatic crests of the maxilla. Mandibular miniplates were inserted first, and after a week maxillary plates were placed under local anesthesia (2% lidocaine with 1:100,000 epinephrine) by a maxillofacial surgeon using the same surgical protocol described by Cevidanes et al.^15^ Three weeks after the surgery, maxillary-mandibular elastics with ¼-in size (Wildlife elastics; American Orthodontics, Wisconsin, USA) between the hooks of the miniplates on both sides were used (Fig 4). A removable appliance with posterior bite blocks was also used to help remove occlusal interferences. The elastics force was measured at 250 g (Fig 5). The patient was instructed to use the intermaxillary elastics 24 hours a day, except when eating or brushing, and to change his elastics every day. After four weeks, a posterior crossbite was starting to develop, so an expansion screw was added to the removable appliance and the patient was advised to open the screw two times a week. After three months the patient had an edge to edge occlusion, and the elastics were changed to 3/16-in medium size elastics. The force delivered by this elastic was 350g. After eight months of orthopedic treatment, patient gained a 2-mm positive overjet and the anterior crossbite was eliminated (Fig 6B). After the active orthopedic therapy, fixed orthodontic treatment began using a fixed MBT (0.022-in) appliance during which elastics were still used for retention until peak height velocity (PHV) was over. Because of the Bolton discrepancy between the anterior maxillary and mandibular teeth, the anterior maxillary teeth were built-up using composite veneer techniques following orthodontic treatment. After twelve months of fixed orthodontic treatment, the ideal dental midline relationship, overjet, and overbite were achieved, and the orthodontic treatment was finished (Fig 7).

### Results

**Figure 8:**
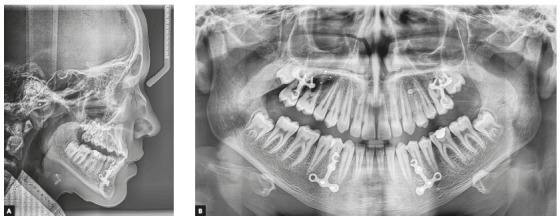
A) Post-orthopedic treatment lateral cephalometric radiograph. B) Post-orthopedic treatment panoramic radiograph.

A 2-mm overjet was attained after eight months of active orthopedic treatment (T_1_). Soft tissue and skeletal relationship were also improved (Fig 6A). Post-orthopedic cephalometric analysis showed that the ANB has been increased by 4.1◦. Maxillary and mandibular incisors were protracted by 1 mm and 1.4 mm, respectively, in relation to their corresponding jaw. Maxillary incisors inclination angle did not change, but mandibular incisors inclination angle (IMPA) increased by 2.8◦. Mandibular angle (SN-GoGn=34◦) was slightly increased (2.1◦) and P-A facial height ratio was decreased by 1.9%. The palatal plan was rotated counterclockwise by 4.8◦ (Fig 8, Fig 10, Table 1).

**Figure 9:**
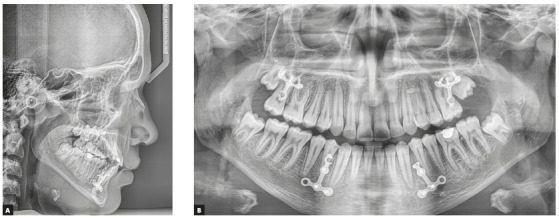
A) Final post-treatment lateral cephalometric radiograph. B) Final post-treatment panoramic radiograph.

**Figure 10:**
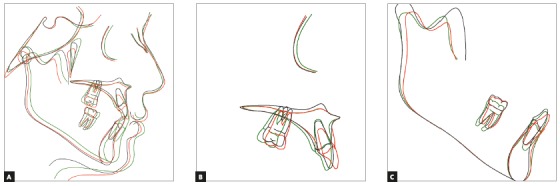
A) Superimposition on anterior cranial base at sella. B) Superimposition at palate. C) Superimposition at mandibular plan. Red = after orthodontic treatment (T_2_); black = after orthopedic treatment (T_1_); green = before treatment (T_0_).

After completing the 12 months fixed orthodontic treatment using 0.022-in appliance with MBT prescription (T_2_), the 2-mm positive overjet was maintained and the posterior open bite was eliminated. The cephalometric analysis after completion of orthodontic treatment showed that the ANB angle was further decreased (by 0.8°) (T_2_vs T_1_) whereas the maxillary and mandibular incisor inclination was increased (U1-SN by 3.2° and IMPA by 5.8°). Mandibular plane angle decreased after fixed orthodontic treatment (by 1.9°). P-A facial height ratio was increased to 64.4% after orthopedic and fixed orthodontic treatment, which was the same as before the orthopedic treatment had even begun (T_0_). These results show that the increased facial height caused by the bi-maxillary plate treatment is temporary and after the fixed orthodontic treatment, facial height ratio and mandibular angles return to the same numbers as before the treatment. The palatal plane was rotated clockwise by 1.1 degrees (Fig 9, Fig 10, Table 1).

## DISCUSSION

In this case, maxillary protraction was achieved with full skeletal anchorage using miniplates in maxilla and mandible. The miniplates used as anchorage for this patient were Y (for maxilla) and T (for mandible) surgical miniplates, that were altered to be able to be used with the elastics. These miniplates were different from the Bollard miniplates used by De Clerk^12^ or the ones made by Sugawara et al.^13^, but no discernible difficulties for usage of elastics by patient and soft tissue irritation could be found. The application of these altered surgical miniplates for orthodontic anchorage could become an option for treating patients in many countries, due to some problems regarding with the availability and costs of these special miniplates. Conventional orthopedic therapy for maxillary protraction (Facemask) is usually performed in early mixed dentition. Treatment results are usually limited and short-lived in patients, and there is a significant chance of relapse and return of reverse overjet once the mandibular growth is finished.^14^ ^,^ ^15^ In this case after the conclusion of orthopedic treatment and during the fixed orthodontic treatment, no change in overjet even during PHV could be detected. Although because of the high mandibular growth during the PHV, 12 months after orthopedic treatment, a small decrease in ANB angle could be detected (from 1.6^o^ to 0.8^o^) which is in accordance with other studies on this protocol.^10^ ^,^ ^13^ ^,^ ^17^ Most of the observed changes with facemask, especially in adolescent patients were due to dental compensations, while skeletal change constituted a small percentage of observed outcome.^5^ ^,^ ^6^ ^,^ ^15^ ^,^ ^16^ Also, patients cooperation and tissue irritation are of concern with the facemask and if the traction is applied through dental anchorage, adverse dental side effects can be observed.^17^ In this case, considering the patient’s age and development, bone-anchored maxillary protraction (BAMP) treatment protocol was chosen because the fully skeletal-based traction can result in stable skeletal maxillary protraction, even in an adolescent patient.^16^

**Figure 11:**
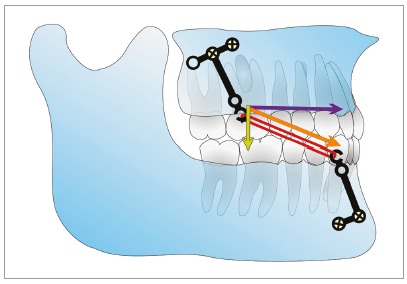
Vector decomposition for the force from intermaxillary elastic. Orange = total force, purple = horizontal component of force, yellow = vertical component of force.

In the discussed case, after the orthopedic treatment, mandibular plane angle was increased and palatal plane had a counterclockwise rotation. This result is similar to other studies.^10^ ^-^ ^13^ ^,^ ^15^ ^,^ ^16^ ^,^ ^18^ ^-^ ^20^ The mandibular plane angle change and the backward rotation of mandible can be attributed to the forward, downward direction of force to the posterior of maxilla and the backward direction of force which is applied to the anterior of the mandible from intermaxillary elastics (Fig 11). The mandibular backward rotation and elimination of forward mandibular shift can be the main reasons behind the improvement of the overjet and ANB observed in this case. The anterior mandibular shift usually results from the functional forward positioning of mandible in patients with edge to edge anterior occlusion (OJ = 0mm). Mandibular shift minimizes the trauma on the mandibular incisors during function. This forward positioning of the mandible will move the B point forward and can decrease the ANB angle. The force direction of the intermaxillary elastics on the mandible is similar to the force direction produced by chin cups and this can produce similar results such as the backward rotation of the mandible. It should be noted that the facial height increase caused by the backward rotation of mandible was eliminated after the fixed orthodontic treatment. Because of this reason, the bone-anchored maxillary protraction can be recommended for treating Class III patients with normal and short faces, and even patients with a mild increase in their facial height. 

Mandibular incisal inclination increased after both orthopedic and fixed orthodontic treatment, which is in line with the findings from other similar studies.^10^ ^-^ ^13^ ^,^ ^16^ ^,^ ^19^ This result could be attributed to the increased tongue pressure after elimination of anterior crossbite and the increased distance between the upper and lower incisors, which in turn allowed the lower incisors to tip forward.

## CONCLUSION

In this case report, a patient with maxillary retrognathia was treated using mini-plate orthopedic treatment method as an alternative to surgery, which was effective in the elimination of the crossbite and helping the patient achieve good facial esthetics. The lower incisor inclination increased unlike the alternative treatment methods and also the achieved overjet was maintained after the orthopedic treatment and during the fixed orthodontic treatment. This treatment protocol was minimally invasive, and the patient’s compliance was maintained throughout the treatment. Utilizing miniplates can be a good alternative to surgery in treating Class III patients with financial and geographical limitations.
